# Machine learning and feature selection for drug response prediction in precision oncology applications

**DOI:** 10.1007/s12551-018-0446-z

**Published:** 2018-08-10

**Authors:** Mehreen Ali, Tero Aittokallio

**Affiliations:** 1 Institute for Molecular Medicine Finland (FIMM), University of Helsinki, FI-00290 Helsinki, Finland; 2 Helsinki Institute for Information Technology (HIIT), Aalto University, FI-02150 Espoo, Finland; 3 Department of Mathematics and Statistics, University of Turku, FI-20014 Turku, Finland

**Keywords:** Precision oncology, Omics profiling, Drug response prediction, Predictive biomarkers, Multi-view regression, Feature selection

## Abstract

In-depth modeling of the complex interplay among multiple omics data measured from cancer cell lines or patient tumors is providing new opportunities toward identification of tailored therapies for individual cancer patients. Supervised machine learning algorithms are increasingly being applied to the omics profiles as they enable integrative analyses among the high-dimensional data sets, as well as personalized predictions of therapy responses using multi-omics panels of response-predictive biomarkers identified through feature selection and cross-validation. However, technical variability and frequent missingness in input “big data” require the application of dedicated data preprocessing pipelines that often lead to some loss of information and compressed view of the biological signal. We describe here the state-of-the-art machine learning methods for anti-cancer drug response modeling and prediction and give our perspective on further opportunities to make better use of high-dimensional multi-omics profiles along with knowledge about cancer pathways targeted by anti-cancer compounds when predicting their phenotypic responses.

## Introduction

Genome-wide genomic profiling approaches based on next-generation sequencing (NGS) of thousands of patient tumors have provided “big data” resources to map the disease mechanisms (The Cancer Genome Atlas, TCGA, http://cancergenome.nih.gov/; International Cancer Genome Consortium, ICGC, https://dcc.icgc.org; Weinstein et al. 2013; Cerami et al. 2012). When combined with clinical information about patient characteristics and treatment outcomes over the course of cancer progression and relapses, such resources enable integrated approaches toward improving both diagnostic and therapeutic options. Compared to conventional clinical management, which treats cancers as homogeneous entities, the “precision oncology” approach seeks to find a molecularly-targeted treatment for each cancer patient sub-type or individual patient (i.e., stratified or personalized medicine, respectively). Matching of available treatment to patients is typically based on somatic aberrations, such as genomic mutations or molecular alterations, provided that therapeutically actionable markers are found and can be used in the clinical practice (Meric-Bernstam et al. 2015). An important pre-requisite for the precision oncology approach is therefore the ability to identify panels of biomarkers associated with the treatment responses in a given patient cohort. If such markers generalize beyond the discovery cohort to new cancer patients, we call them here as “predictive biomarkers.”

Traditional analytical strategies for finding treatment response-associated markers typically start from unsupervised clustering of the molecular and/or genomic profiles of the patient samples, and then subsequently try to identify the treatments showing therapeutic efficacy in the distinct sample sub-clusters (Hoadley et al. 2014; Campbell et al. 2017). The use of other clinical outcomes, such as prognostic classes in thousands of patients, can also enable the detection of statistically associated multi-gene interactions in genomic-based studies, which can be restricted to a single cancer type (Papaemmanuil et al. 2016). Alternatively, one can start from the treatment response clustering and then go into the genomic or molecular correlates that explain the observed drug sensitivity or resistance clusters of patient-derived samples (Pemovska et al. 2013; Tyner et al. 2013; Frismantas et al. 2017; Andersson et al. 2018). However, studies that focus on single cancer type and/or multiple treatment profiles are often underpowered for multi-marker statistical regression of drug response patterns. The paucity of available clinical treatment information for the profiled patient tumors also complicates the biomarker discovery task. Besides a few notable cases of clinical success, single markers, regardless whether they come from somatic mutations or other omics data, are not generally accurate enough for explaining treatment responses for most drug classes (Nguyen et al. 2016); instead, multivariate modeling in larger sample sizes is required for identifying reliable marker panels (Dietrich et al. 2018).

Supervised machine learning models offer the opportunity for multi-marker prediction of drug responses using multi-omics and multi-task learning approaches that leverage information across both patient samples as well as across drug similarities (Costello et al. 2014; Ammad-ud-din et al. 2016; Cichonska et al. 2018). However, the accuracy of such machine learning models depends critically on the availability of high-quality training data from large-enough sample sizes. Therefore, most of the learning studies to date have been done using large panels of cancer cell lines (Garnett et al. 2012; Barretina et al. 2012; Seashore-Ludlow et al. 2015; Iorio et al. 2016), although there are also a few recent examples aiming at clinical treatment predictions in patient samples (Sadanandam et al. 2013; Geeleher et al. 2014; Majumder et al. 2015; Ding et al. 2016; Noren et al. 2016; Yao et al. 2018; Turki et al. 2018). Rather than conducting a systematic review of all the works published on this broad topic (please see, e.g., Azuaje 2017), we describe below application cases of supervised machine learning models to drug response prediction in cancer cell lines and discuss to which extend these models could be also applied in a clinical setting to individualized treatment selection once large-enough patient cohorts become available. We also critically evaluate whether the current learning approaches benefit from the use of “big-scale” omics data, which still mainly originate from the NGS-based technologies, and provide our perspective on the future directions required for supporting clinical applicability, both in terms of improved modeling frameworks and most informative omics measurements used as input for these models.

## Drug sensitivity prediction challenge

The selected machine learning models and omics measurements are described and compared here in the context of the DREAM Challenges, which provide systematic and objective means to assess the predictive power of the models and measurements by means of large-enough validation datasets that are hidden to the Challenge participants, and therefore can be used as independent test data. The Challenges organized by the Dialogue for Reverse Engineering Assessment and Methods (DREAM, http://dreamchallenges.org/) implement a community-based crowdsourcing solution for complex questions in biology and medicine, through collaborative competitions and open-data sharing, hosted by the Sage Bionetworks (http://sagebase.org/). The primary focus here is on the NCI-DREAM7 Drug Sensitivity Prediction Challenge (Costello et al. 2014), but we also extend to newer machine learning models introduced after the Challenge, especially those that also implement feature selection techniques to identify such combinations of genomic and other features from the multi-omics profiles that are most predictive of the drug response phenotypes. Such predictive panels of biomarkers are critical for clinical translation.

### The Challenge setup and winning model

The DREAM7 Challenge, organized together with the National Cancer Institute (NCI), specifically targeted the development and benchmarking of drug sensitivity prediction algorithms, as a stepping stone toward precision oncology (Costello et al. 2014). We use here this NCI-DREAM Challenge, as well as a number of follow-up studies conducted by us and others, to demonstrate the lessons learned from the use of machine learning models for drug response prediction, and especially the importance of high-dimensional omics profiles in such prediction problem. In NCI-DREAM7 Challenge, the training of the prediction algorithms was based on genome-wide omics profiles of 53 human breast cancer cell lines. These omics profiles included large-scale somatic DNA copy number variation (CNV), DNA methylation, and point mutations, along with transcript expression, RNA-sequencing, and protein abundance profiled from untreated cells (Table 1). The drug treatment sensitivity was measured using pGI_50_ readout (–log_10_GI_50_, where GI_50_ refers to the drug concentration required to inhibit 50% of maximal cell growth), in response to 28 anti-cancer therapeutic compounds. The aim of the NCI/DREAM7 Challenge was to predict the drug responses (sensitivity or resistance) of the test cell lines to the same or similar compounds using various statistical and machine learning models.

**Table 1:** Details of the key omics datasets available from representative cancer cell lines and patient genomic resources, along with the dataset sizes and dimensionalities of the raw and processed profiles for the NGS-based datatypes (rows in italics). For the other datatypes, only the dimensionality of the processed data is reported for comparison

	NCI-DREAM7^1^	NCI-60^2^	GDSC1000^3^	TCGA^4^/TCPA^5^
Cancer type	Breast cancer	9 tissue types	29 tissue types	33 tissue types
Number of samples	53 cell lines	59 cell lines	1124 cell lines	~ 11,000 patient tumors
*Total size of NGS datatypes (raw datasets)*	***~ 13 TB***	***~ 15 TB***	***260 TB***	***~ 2.5 PB***
*Whole genome sequencings (~ 3.2 billion reads/sample)*	*~ 8 TB*	*~ 9 TB*	*~ 170 TB*	*~ 1.6 PB*
*Whole exome sequencings (150 million reads/sample)*	*~ 4.5 TB*	*~ 5 TB*	*~ 90 TB*	*~ 0.8 PB*
*RNA sequencing (30–100 million reads/sample)*	*~ 55 GB*	*~ 60 GB*	*~ 1 TB*	*~ 11 TB*
*MicroRNA profiles*	*–*	*~ 5 GB*	*–*	*~ 500 GB*
Total size (processed datasets)	**~ 27 GB**	**~ 33 GB**	**~ 90 GB**	**~ 480 TB**
Whole genome sequencing	~ 20,000 genes	~ 17,000 genes	19,100 genes	~ 21,000 genes
Whole exome sequencing	~ 22,000 genes	~ 13,000 genes	~ 23,000 genes	~ 20,000 genes
RNAsequencing	~ 40,000 transcripts	~ 60, 000 transcripts	~ 50, 000 transcripts	~ 55,000 transcripts
MicroRNA profiles	–	~ 800 miRNA transcripts	–	~ 1800 miRNA transcripts
Microarray gene expression	~ 18,000 genes	25,722 genes	17,737 genes	~ 22,000 genes
Somatic mutation calling	~ 33,000 SNPs^6^	~ 500,000 SNPs	~ 485,000 SNPs	~ 500,000 SNPs
Copy number variation	~ 27,000 variants	~ 25,000 variants	~ 50,000 variants	~ 50,000 variants
DNA methylation patterns	~ 27,000 CpGs^7^	20,000 CpGs	~ 35,000 CpGs	~ 486,000 CpGs
RPPA^8^ proteomics	131 proteins	162 proteins	–	~ 240 proteins
MS^9^ proteomics	–	10,350 proteins	–	~ 16,000 proteins^10^
Drug response data	28 compounds	> 100,000 compounds	265 compounds	Survival data for clinical treatments

Boldface entries represent total sizes of raw and processed datasets. These are not statistical significance values

^1^*NCI-DREAM7*, DREAM7 Challenge (http://dreamchallenges.org/), organized together with the National Cancer Institute (NCI; Costello et al. 2014)

^2^*NCI-60*, The National Cancer Institute drug screening panel (Shoemaker 2006)

^3^*GDSC1000*, Genomics of Drug Sensitivity in Cancer project (Yang et al. 2012)

^4^*TCGA*, The Cancer Genome Atlas (http://cancergenome.nih.gov/; Weinstein et al. 2013)

^5^*TCPA*, The Cancer Proteome Atlas (http://tcpaportal.org/tcpa/, Li et al. 2013)

^6^*SNPs*, single-nucleotide polymorphism

^7^*CpGs*, CpG island in DNA where “C” is connected to “G” by a phosphodiester bond “p”

^8^*RPPA*, reverse phase protein array

^9^*MS*, mass spectrometry

^10^*CPTAC*, Clinical Proteomic Tumor Analysis Consortium (https://proteomics.cancer.gov/programs/cptac)

Over 65 teams participated in the Challenge and tested various combinations of the 6 omics profiles, along with multiple approaches to deal with missing data inherent to the high-throughput measurements (e.g., imputation methods), and/or incorporating a prior biological knowledge in the form of annotated biological pathways from KEGG (Kanehisa and Goto 2000) or MSigDB (Liberzon et al. 2011). Interestingly, the predictive models using all the omics profiles had the best performance among all model submissions, suggesting that the genomic, epigenomic, and proteomic profiles provide complementary signal for the drug response prediction. Subsequent analysis of the 44 prediction algorithms highlighted the importance of modeling nonlinear relationships, along with the use of a prior biological knowledge of the breast cancer oncogenes and disease-driving pathways.

The best-performing approach was based on the Bayesian efficient multiple kernel learning (BEMKL) model (Gönen 2012), a kernelized regression model that makes use of multi-task and multi-view learning (Costello et al. 2014). In the winning BEMKL model, the pairwise similarities of cell lines in terms of the multiple omics profiles are represented as separate profile kernels. Multiple kernel learning (MKL) algorithm (Gönen and Alpaydın 2011) then calculates a combined kernel as the weighted sum of all profile-specific kernels. Multi-task learning (MTL), on the other hand, allows BEMKL to train the model simultaneously for all the drugs (tasks). BEMKL first estimates drug-specific intermediate variables for each kernel through Bayesian inference using cell line weights and then estimates the output drug response matrix by sharing the kernel weights across all the drugs. Hyper-parameters and an error term (also known as bias) are introduced in BEMKL to account for the modeling bias arising due to the varying rate of missing values and noise in the drug response data set.

More specifically, BEMKL model predicts drug response of an unseen cell line *x*_∗_ based on a single omics profile *X* ∈ *ℝ*^*N* × *D*^, where *N* is the number of cell lines and *D* is the number of features. This regression problem can be formulated by the following kernel-based decision function:

1 $$ f\left({x}_{\ast}\right)={a}^T\ {k}_{\ast }+b $$

where *k* : *X* × *X* ⟹ *ℝ* is profile-specific kernel, *a* represents unknown weights for cell lines, and *b* stands for drug-specific bias. There is a need to introduce distributional assumptions for *a* and *b*, in order to model nonlinear relationships between cell lines using kernelized regression formulation in Eq. 1:

$$ {\displaystyle \begin{array}{ll}{\lambda}_{t,n}\sim \mathcal{G}\left({\lambda}_{t,n};{\alpha}_{\lambda },{\beta}_{\lambda}\right)& \forall \left(t,n\right)\\ {}{a}_{t,n}\sim \mathcal{N}\left({a}_{t,n};0,{\lambda}_{t,n}^{-1}\right)& \forall \left(t,n\right)\\ {}{\gamma}_t\sim \mathcal{G}\left({\gamma}_t;{\alpha}_{\gamma },{\beta}_{\gamma}\right)& \forall (t)\\ {}{b}_t\sim \mathcal{N}\left({b}_t;0,{\lambda}_t^{-1}\right)& \forall (t)\end{array}} $$

Here, $\mathcal{N}\left(;\mu, \Sigma \right)$ and $\mathcal{G}\left(;\alpha, \beta \right)$ refer to normal and gamma distributions with *μ* mean, Σ covariance, *α* shape parameter, and *β* scale parameter, respectively, and *t* represents drug response prediction of a single drug as a single task. The interested reader is referred to Gönen (2012) and Costello et al. (2014) for more detailed explanation of Bayesian formulation of BEMKL model for multiple omics profiles, along with the distributional assumptions and specific constraints applied. Importantly, the joint MT-MKL strategy yields an increased signal-to-noise ratio in the heterogeneous and noisy omics datasets and therefore improves predictive power for drug sensitivity predictions.

### Lessons learned and further model developments

It has been observed in the DREAM7-NCI Challenge and in other studies that most of the variability in the drug response levels across the cell lines can be explained by the genome-wide gene expression data, whereas the other omics profiles only marginally improve the prediction performance (Jang et al. 2014; Costello et al. 2014). However, the use of multiple omics profiles from various biological levels can still improve the prediction results (Cortés-Ciriano et al. 2016), especially in the case of small sets of samples (cell lines or patient sample) and/or feature profiles (genes or drugs) (Amin et al. 2014). Therefore, the development of drug response prediction models critically depends on the input data type, dimensionality, noise ratio, data heterogeneity, and complexity along with the particular prediction problem. To decrease the dominance of the gene expression profiles, which sometimes make the interpretation of the prediction models difficult, Aben et al. (2016) developed a two-stage approach, called TANDEM, which first explains the drug responses using point mutations, copy number variation, methylation, and cancer type and only in the second stage explains the remaining variability in the response levels using gene expression. Predictive models can also be tailored, for instance, to specific cancer type or drug classes, or alternatively, one may choose a pan-cancer approach, to model multi-drug class or even combinatorial drug response prediction as problem domain (Menden et al. 2018; Aben et al. 2018). Typical large-scale data preprocessing includes noise filtering, feature engineering, and normalization, which are particularly important for high-throughput measurement data from a limited sample size for model training. In addition to the training data volume and quality, the performance of predictive models has also shown to depend on the given drug sensitivity measure and statistical indicator (Azuaje 2017).

In our recent work (Ali et al. 2018), we investigated the relative contribution of various omics profiles, focusing especially on the proteomics profiling for the drug sensitivity prediction in the NCI-60 pan-cancer cell line data (Shoemaker 2006). The NCI-60 cell line panel comprises of 60 cell lines spanning over 9 cancer types, which are tested against ~ 15,000 anti-cancer therapeutics (Table 1). Multiple omics profiles are publicly available for these cell lines, including global mass spectrometry (MS)-based proteomic profiling (Gholami et al. 2013). An integrated BEMKL model based on the multi-omics profiling improved drug sensitivity prediction as compared to the prediction performance solely based on gene expression profile. Notably, although the global MS proteomic data includes a total of 8113 proteins, the NCI-60 cell lines contains, on average, 55% missing proteomic data, which greatly complicates the predictive modeling. After considering only the completely measured proteins in MS-based profile, the predictive performance was increased significantly for molecularly-targeted drugs as compared to using MS data alone with all the proteins. However, considering only the completely measured census cancer genes from COSMIC (http://cancer.sanger.ac.uk/cosmic) for the MS and other omics profiles surprisingly improved drug response predictions for 75% of the NCI-60 drugs for both sets of selected 47 cytotoxic and 24 targeted drugs, separately. Interestingly, this filtering reduced the MS data from 8113 proteins to 42 proteins, yet leading to statistically significant improvements. We further tested a number of general imputation methods as well as ones tailored for MS data (Webb-Robertson et al. 2015), but these did not improve the prediction results as much as the data filtering (Ali et al. 2018).

### Use of auxiliary information and feature selection

Auxiliary information for drugs or cell lines has also been shown to boost the predictive power by providing a prior information for the drug response prediction (Ammad-ud-din et al. 2016; Yang et al. 2018). For instance, component-wise MKL (cwKBMF) identifies groups of output variables (drug responses) and applies MKL on such subsets of auxiliary side information (cell line information as omics profiles and drug information as drug chemical properties) in a Bayesian setting (Ammad-ud-din et al. 2016). In the NCI/DREAM7 Challenge, the BEMKL method demonstrated improved predictive performance as it enabled capturing common signal among multiple omics profiles, resulting in an increased signal-to-noise ratio (Costello et al. 2014). Similarly, cwKBMF provides an opportunity to add additional side information on drugs (such as biological, chemical and/or structural information). Compared to BEMKL, cwKBMF further refines the use of a prior biological knowledge for subsets of multiple side-data views, including biological pathway information, hence enabling one to infer target or other pathways associated with the drug’s mode-of-action (Ammad-ud-din et al. 2016).

Transfer learning (TL; Turki et al. 2018) is another way of incorporating auxiliary information among different cancer cell lines to improve in vitro or in vivo drug sensitivity prediction. Training data in TL consists of expression profile and drug responses of tissue-specific training cell lines/samples as well as of cell lines/samples of related tissue types (added as auxiliary data), whereas the test data consists of expression profile of tissue-specific test samples only. This method is useful even when data distributions and feature space of tissue-specific and auxiliary data differ. Transferring knowledge from auxiliary data to training cell lines involves three steps: (i) shifting representation of auxiliary data to match the training data using a modified version of Gaussian blurring mean shift (GBMS; Wang and Carreira-Perpinán 2010), (ii) optimal alignment between training and auxiliary data using procrustes analyses (Wang and Mahadevan 2008), and (iii) applying standard machine learning method (e.g., support vector regression with linear or sigmoid kernel ridge regression and logistic ridge regression) to learn a highly accurate in vivo or in vitro model. Trained model is then applied on test data to make in vivo response predictions.

There are also a number of supervised machine learning methods that implement detailed feature selection to learn the panels of genomic or molecular features (e.g., gene or protein expression changes combined with somatic alterations) that are most predictive of the drug response (see Table 2 for examples). In addition to general feature selection techniques, such as elastic net or random forests, that can be applied to any high-dimensional dataset, researchers have also developed novel feature selection techniques specifically for drug response prediction problem. Many of these techniques employ a prior information about the MoA of the drugs, such as their protein targets or biological pathways (Ammad-ud-din et al. 2017; Yang et al. 2018). Such response predictive panels of multi-omics biomarker are critical for clinical translation of the modeling results to new patient cohorts.

**Table 2:** Representative drug sensitivity prediction models classified in terms of whether or not they implement also feature selection

	Prediction model	Example applications
Kernel-based	SVM^1^	Dong et al. (2015) used SVM classification model to predict drug sensitivity accurately for several drugs using baseline gene expression of cell line panels from preclinical studies (CCLE^2^ and CGP^3^) as features. Other applications of SVM for drug response prediction include, e.g., Costello et al. (2014), Jang et al. (2014), and Hejase and Chan (2015).
	BEMKL	Kernelized regression model for drug response prediction based on data integration across multiple omics profiles, through multi-task, multiple kernel learning (Costello et al. 2014; applications in breast cancer cell line panel). A particular emphasis was placed on the proteomic profiles in our follow-up work using NCI^4^-60 human tumor cell lines screen (Ali et al. 2018).
	cwKBMF^5^	Drug response prediction model (Ammad-ud-din et al. 2016) by utilizing cell line information along with the drug chemical properties as an additional information source through selective data integration. Applications in GDSC^6^ and CTRP^7^ cancer cell line panels, and wet-lab validations in AML cell lines conducted in-house.
	KRL	Kernelized rank learning (KRL; He et al. 2018) is a personalized drug recommendation method that selects the most promising drug based on its predicted effect per cell line. Applications shown in GDSC cell lines and TCGA breast cancer patients using one expression profile at a time.
Feature selection-based	Ridge Regression	Geeleher et al. (2014) and Geeleher et al. (2017) applied ridge regression model to predict drug responses in GDSC cell lines, and inferred marker panels for predicting comprehensive drug response profiles in patient tumors in the TCGA dataset (Geeleher et al. 2017).
	Elastic net	Jang et al. (2014) found elastic net regression as one of the best-performing modeling strategies for drug response prediction in CCLE and GDSC cancer cell lines. Similarly, Ding et al. (2018) applied elastic net regression to generate logistic models for drug sensitivity prediction through deep learning in CCLE and GDSC datasets.
	Random forests	Riddick et al. (2010) built an ensemble regression model using random forest (RF) for drug sensitivity prediction in NCI-60 cell line panel. The model was also used to create drug-specific gene expression signatures and identify core cell lines associated with each drug’s response. Other applications of RF include, e.g., Menden et al. (2013), Nguyen et al. (2016), and Rahman et al. (2017).
	MVLR^8^	Bayesian multi-view multi-task linear regression model (Ammad-ud-din et al. 2017) for drug response prediction by capitalizing on feature combinations that are most predictive of the drug’s response. This method also enables one to use functional-linked-networks (FLNs) as prior biological knowledge. Applications in GDSC and in-house TNBC^9^ cell line panels.

^1^*SVM*, support vector machines

^2^*CCLE*, Cancer Cell Line Encyclopedia

^3^*CGP*, Cancer Genome Project

^4^*NCI*, National Cancer Institute

^5^*cwKBMF*, component-wise kernelized Bayesian matrix factorization

^6^*GDSC*, Genomics of Drug Sensitivity in Cancer project

^7^*CTRP*, Cancer Therapeutic Response Portal

^8^*MVLR*, multi-view linear regression

^9^*TNBC*, triple-negative breast cancer

### Model applications to cancer patient cohorts

In addition to the cancer cell line panels, genomic and molecular profiling has also been performed in patient tumor samples. For instance, TCGA provides a comprehensive cohort of omics and clinical information, across 33 different cancer types, consisting of genomic, molecular, proteomic, and clinical features of > 11,000 cancer patients, with the aim to enhance understanding of cancer mechanisms for improved diagnosis and treatment options. The number of patients for each tissue type ranges from 36 to 1100 (Table 1). Although survival times and other clinical endpoints are available for those treatments the patients were given over the course of their clinical management, these samples have not been subjected to a large-scale drug sensitivity profiling using laboratory assays.

To that end, Geeleher et al. (2017) recently developed a novel computational method that allows one to computationally impute drug response in large clinical cancer genomics data sets such as the TCGA. Their approach first trains linear ridge regression models described before (Geeleher et al. 2014; see Table 2), through linking gene expression to drug response in large panels of cancer cell lines from the GDSC1000 resource (Table 1), and then applies the estimated models to the batch-corrected tumor gene expression data in the TCGA data. This provides an “imputed drug response profile” for each patient over 138 drugs. Their approach was able to re-construct some known drug associations for clinically actionable somatic genetic alterations, along with identifying novel predictive biomarkers for investigational compounds and approved drugs that require further clinical validation.

Similarly, Turki et al. (2018) showed that TL improves drug response predictions of tissue-specific clinical trial samples by transferring knowledge from auxiliary data of related cell lines or tumor samples. Due to many biological differences between cell lines and patient primary tumors, the clinical applications of the machine learning models to predict patient’s treatment responses in vivo will ideally require training and careful testing of the models in large-enough patient cohorts. An example experimental setting would be applying TL model trained on the set of multiple myeloma patients combined with breast cancer auxiliary data and predicting drug sensitivities of multiple myeloma patients, or applying TL on training set of non-small cell lung cancer patients with triple-negative breast cancer auxiliary data to test response predictions of a set of non-small cell lung cancer patients. TL can also make use of patient electronic health records to transfer knowledge.

## Conclusions and future directions

Based on the lessons learned from the DREAM Challenges and other related benchmarking studies, the NGS-based “big data” is not yet among the most predictive genomic or molecular features for drug response prediction globally, except for the few known examples of cancer types that are driven by single somatic aberrations, such as BCR-ABL-positive chronic myeloid leukemia, non-small cell lung cancer or BRAF in melanoma, with clinically actionable small-molecule inhibitors available (Flaherty et al. 2012; Pemovska et al. 2015). With regard to other cancer or drug classes, which especially require multi-marker panels, the microarray-based gene expression and targeted protein abundance profiles appear currently as the most predictive source of signal (Costello et al. 2014). This is likely due to the fact that these profiling platforms have been around for some time already, and available tailored processing methods have been developed for these. For the more recent NGS-based platforms, such as DNA copy number or point mutations, we are still lacking the knowledge of how to best utilize all the hidden nuggets of information available from the raw sequencing data for drug response prediction; instead, one needs to rely only on the most processed, limited “gene-level” data available (Table 1). The same applies to some extend also to genome-wide RNA-seq transcriptomics and especially to the MS-based global proteomic profiling, which would benefit from standardized analytical approaches to extract more accurate and complete gene expression and protein activity profiles. For example, our pilot study showed that the MS-based proteomics can significantly improve the drug response predictions, but only after filtering out most of the protein measurements (Ali et al. 2018). Similarly, a recent transcript-level machine learning work demonstrates how the RNA-seq technology offers additional predictive signal, when compared to gene-level expression or mutation information (Safikhani et al. 2017). Therefore, we argue that we will need improvements both in the computational methods and in the experimental assays in order to convincingly show the added value of “big data” for drug response prediction.

Future developments in the machine learning models should therefore be directed toward better use of the integrated and full information from the multiple omics datasets. For instance, how to deal with the redundancy between the predictive profiles in case of anti-correlations between CNV and somatic point mutations, which are widely observed both in tumor samples and in cancer cell lines (Ciriello et al. 2013; Iorio et al. 2016). Based on multiple lines of evidence and benchmarking (Saez-Rodriguez et al. 2016; Guinney et al. 2017), clinical data from the cancer patients, including their standard laboratory tests and other patient characteristics, seems often to provide most predictive signatures for treatment responses (Ding et al. 2018). Even though this is somewhat disappointing from the “precision oncology” perspective, this is not too surprising given that these clinical data have been used for decades by the medical doctors for both diagnostic and treatment selection purposes. The next challenge for the computational community is therefore to show how to improve the prediction accuracies beyond that based on clinical information only, through using all the modern high-throughput biotechnologies such as genomics, proteomics, and metabolomics (Peddinti et al. 2017). In case the sequencing data proves not to be alone sufficient for drug response prediction, then other, even more high-dimensional data sources, such as biomedical imaging or immune-profiling, might provide the necessary level of resolution required for the next leap for treatment selection (Friedman et al. 2015; Horvath et al. 2016). It has been shown with other related applications, including bioimage analysis (Janowczyk and Madabhushi 2016; Wang et al. 2017) and compound-target interaction prediction (Ma et al. 2015; Xu et al. 2017) that when the feature spaces are large enough, deep-learning machine learning models can learn the most predictive signal from such “big data,” without the need of any processing of filtering steps, hence providing opportunity for significant improvements in precision oncology in terms of both treatment response prediction accuracy as well as resources and time required for data processing (Camacho et al. 2018; Chang et al. 2018; Ding et al. 2018).

From the medical point of view, however, rather than thinking what and how much can be measured on a large scale, one should also consider what is the source of information that is most useful for the particular prediction task. For systematic mapping of compound-target interactions, instead of generating more and more compound-target bioactivity data, a more effective approach might be to train machine learning models based on the existing data and then use these models to predict what parts of the massive compound-target universe one should experimentally explore in order to get most benefit from the expensive laboratory experiments (Azencott et al. 2017; Ding et al. 2018). The same approach should be useful also for drug response prediction task, where we already have large-scale data in cancer cell lines, and hopefully soon also in patient samples, to start making more comprehensive machine learning exercises to prioritize the next phases of experimentation. We argue that such data-driven predictive approach will be more cost-effective, compared to the exhaustive approach of sequencing everything, which has been the dominating approach so far in many international efforts. Collection and integration of the already available data are by no means straightforward, requiring both infrastructure developments and common standards for integrating and sharing data from various experimental assays and laboratories. However, such community-based approach will likely provide not only a cost-effective but also a faster track to new biomedical discoveries, as it can also collect large-enough patient cohorts for single cancer types, hence avoiding the need for pan-cancer approaches that may miss important cancer-specific findings. For clinical translation, feature selection remains a critical part of precision oncology as large-scale profiling of each cancer patient is not likely to be possible within the coming years, rather the treatment selection will be based on targeted assays of most predictive markers for a given cancer type.
